# A Rare Presentation of Laurence-Moon-Bardet-Biedl Syndrome: Atypical Retinitis Punctata Albescens and Non-alcoholic Fatty Liver Disease

**DOI:** 10.7759/cureus.54064

**Published:** 2024-02-12

**Authors:** Meghavi Pandya, Sachin Daigavane

**Affiliations:** 1 Ophthalmology, Jawaharlal Nehru Medical College, Datta Meghe Institute of Higher Education and Research, Wardha, IND

**Keywords:** genetic counseling, non-alcoholic fatty liver disease (nafld), central obesity, polydactyly, retinitis punctata albescens, laurence-moon-bardet-biedl syndrome

## Abstract

This case report emphasizes the varied clinical features of Laurence-Moon-Bardet-Biedl syndrome (LMBBS) in a 10-year-old girl, presenting a rare combination of atypical retinitis punctata albescens, polydactyly, central obesity, and non-alcoholic fatty liver disease (NAFLD). Despite extensive management efforts, the patient's visual impairment remained unchanged, highlighting the challenging and progressive nature of LMBBS, particularly its ocular manifestations. Genetic counseling played a crucial role, stressing the significance of early genetic analysis in consanguineous marriages for anomaly detection and informed family planning. This case enhances our comprehension of LMBBS and emphasizes the necessity for ongoing research and multidisciplinary care to tackle its complexities.

## Introduction

Laurence-Moon-Bardet-Biedl syndrome (LMBBS) is a rare and complex autosomal recessive disorder characterized by a constellation of clinical features, including retinal dystrophy, obesity, polydactyly, intellectual disability, and other systemic manifestations [1]. First described by Laurence and Moon in 1866, the syndrome was later expanded by Bardet and Biedl in the early 20th century, recognizing its genetic basis and multi-organ involvement [2]. LMBBS is primarily associated with mutations in multiple genes involved in ciliary function. The proteins encoded by these genes play crucial roles in the structure and function of primary cilia, cellular structures implicated in various developmental processes, and signal transduction pathways. Mutations in these genes disrupt ciliary function, leading to the diverse clinical manifestations observed in LMBBS [3].

The genetic heterogeneity of LMBBS involves several known genes, including BBS1, BBS2, BBS4, BBS5, BBS7, and BBS9, among others. These genes are part of the BBSome complex, which regulates ciliary trafficking and signaling [4]. The varied phenotypic expression in LMBBS is attributed to these genes' wide range of mutations and the complex interactions involved in ciliary function [5]. The classical clinical features of LMBBS include retinal dystrophy, typically presenting as retinitis pigmentosa, obesity, intellectual disability, and polydactyly. However, the syndrome is known for its considerable clinical variability, with additional manifestations affecting the renal, cardiovascular, and endocrine systems [6].

Ocular involvement in LMBBS often manifests as retinitis pigmentosa, leading to progressive visual impairment. The early onset and severity of retinal degeneration contribute significantly to the morbidity associated with the syndrome. However, the present case introduces an atypical retinal manifestation, retinitis punctata albescens, emphasizing the diversity of ocular phenotypes within LMBBS [7]. Metabolic complications, including dyslipidemia and non-alcoholic fatty liver disease (NAFLD), have been reported in individuals with LMBBS. Dyslipidemia, characterized by alterations in lipid profiles, is a common finding and may contribute to the increased risk of cardiovascular disease in affected individuals [8]. While less commonly documented, NAFLD underscores the importance of considering the multisystemic impact of LMBBS [9].

Consanguineous marriages have been identified as a risk factor for the occurrence of autosomal recessive disorders, including LMBBS. Genetic counseling is pivotal in educating families about the potential risks associated with consanguinity, discussing the probability of genetic disorders, and guiding them through the diagnostic and management processes [10].

## Case presentation

**Figure 1:**
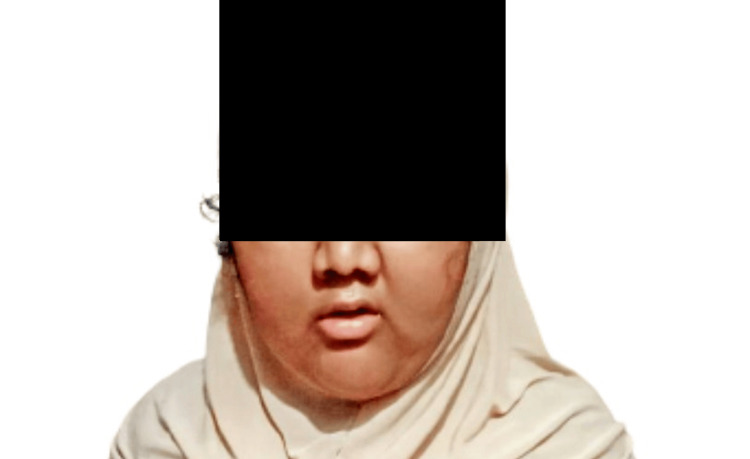
Moon-like face of the patient with LMBBS LMBBS: Laurence-Moon-Bardet-Biedl syndrome

**Figure 2:**
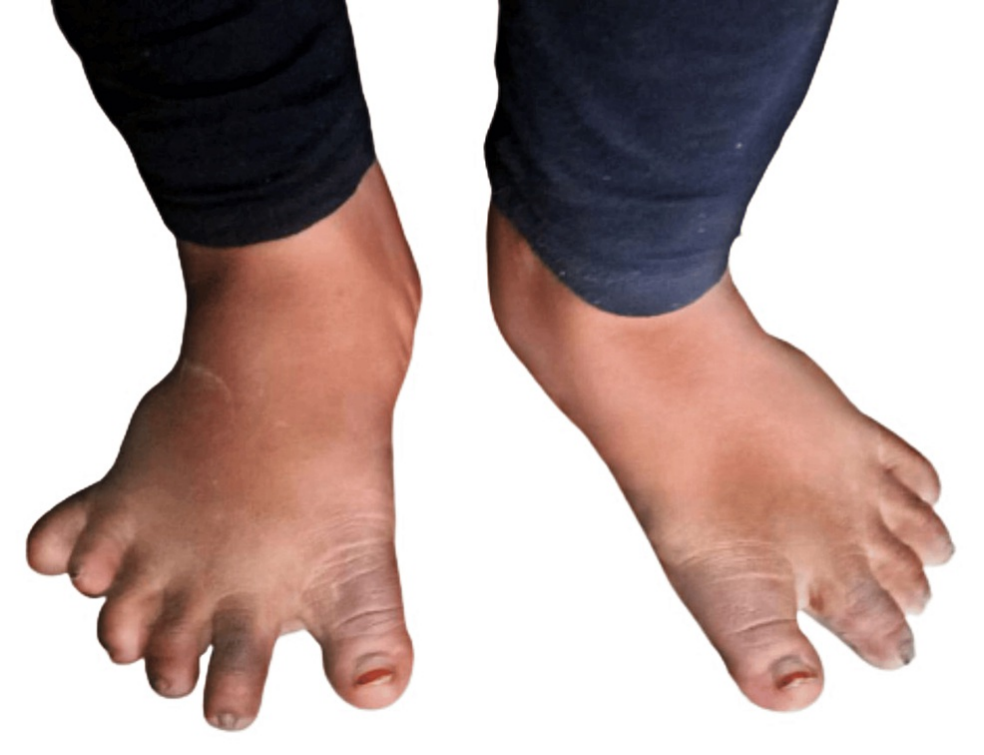
Polydactyly in the right foot

A 10-year-old girl presented at the hospital, primarily complaining of persistent poor vision for the past 2.5 years. Her medical history revealed that she was the first child of a consanguineous marriage and her full-term pregnancy was unremarkable. At three months old, her father reported a progressive weight gain, language delay, milestone delays, and swallowing difficulties. There was no confirmed family history of the ailment. Upon general physical examination (Figure 1), the patient exhibited subnormal mentality, central obesity, a moon-like face, and several dysmorphic features, including a narrow forehead, frontal bossing, low hairline, depressed nasal bridge, upturned nostrils, and long eyelashes. Hand and foot examination revealed polydactyly and syndactyly (polysyndactyly) on the right foot (Figure 2) (Table 1).

**Table 1:** Modified diagnostic criteria for BBS representing major and minor criteria KOT: keratocystic odontogenic tumor; BBS: Bardet-Biedl syndrome

Sr. no.	Major criteria	Present (+) or absent (-)	Minor criteria	Present (+) or absent (-)
1	>2BCC or 1BCC in a patient less than 20 years old	-	Macrocephaly	+
2	KOT	-	Congenital malformation (frontal bossing, cleft lip, and cleft palate)	+
3	>3 palmar or plantar pits	+	Other skeletal anomalies (Sprengel deformity, syndactyly)	+
4	Ectopic calcification or early calcification of the falx cerebri	-	Ovarian and cardiac fibroma or medulloblastoma	-
5	Bifid, fused, or splayed ribs	-	Radiologic abnormalities (vertebral, bridging of the sella turcica)	-
6	First-degree relative affected	-		

**Figure 3:**
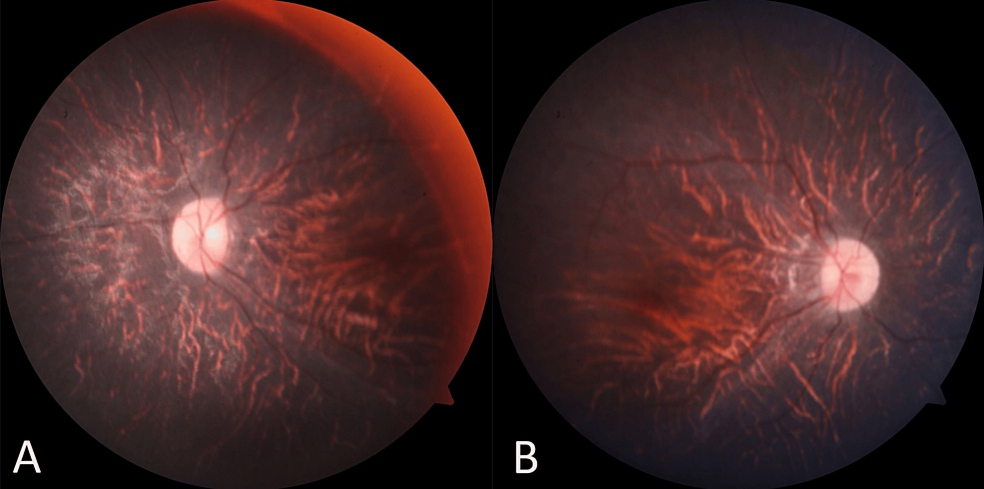
A and B: Fundus photo showing evidence of retinitis punctata albescens with a pale disc and arterial attenuation

**Figure 4:**
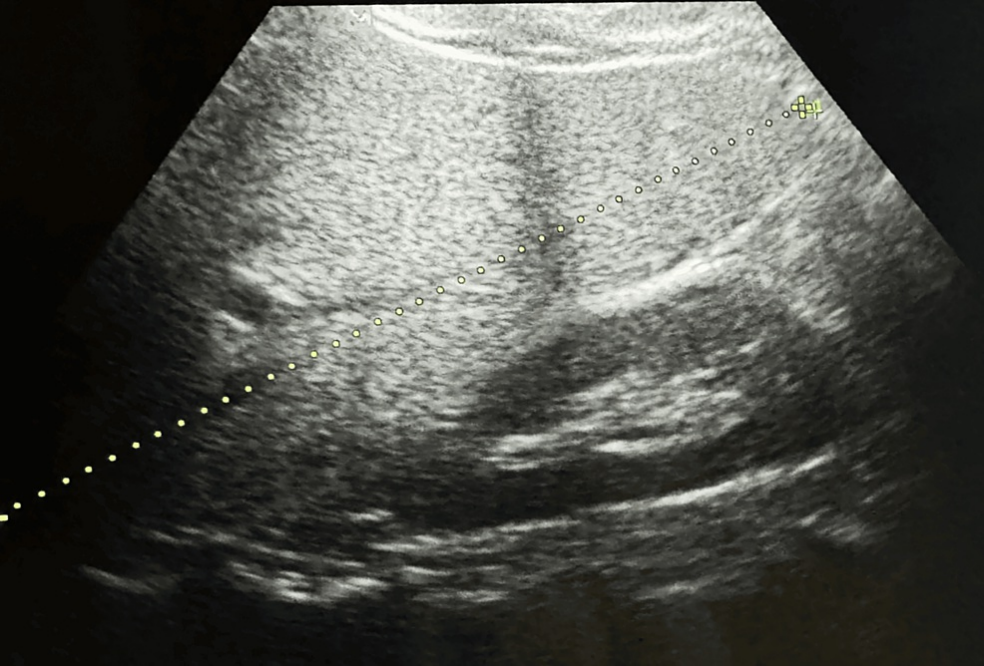
Ultrasonography indicating slightly higher echogenicity than the renal cortex, suggesting grade 1 fatty liver

Ophthalmic assessment revealed poor vision, counting fingers up to 1 meter with no improvement using a pinhole in both eyes. Further, the ophthalmological assessment indicated bilateral atypical retinitis pigmentosa, termed "retinitis punctata albescens," with a pale disc and arterial attenuation (Figure 3A and Figure 3B). A basic lab workup, including a lipid profile, showed lower high-density lipoprotein (HDL) was 31 mg/dL (reference: >40 mg/dL) and low-density lipoprotein (LDL) was 87 mg/dL (reference: <100 mg/dL), while total cholesterol, triglycerides, and very-low-density lipoprotein (VLDL) were normal. The patient's body mass index (BMI) was 33.33 (classified as obese). Ultrasonography indicated slightly higher echogenicity than the renal cortex, suggesting grade 1 fatty liver (Figure 4).

During genetic counseling, the potential complications of consanguineous marriage in children were discussed with the parents. The family was counseled about the possibility of future complications. Genetic analysis was recommended to detect early anomalies, provide appropriate treatment, and significantly slow symptom progression. The patient was prescribed glasses for refraction and advised to follow a diet restricted in fats and refined carbohydrates. A high-protein, low-carbohydrate diet was suggested, along with vitamin A tablets and a multivitamin supplement. Exercise was also recommended, with a follow-up scheduled after three months. Regular pediatric follow-ups were advised to assess developmental growth. Unfortunately, no improvement in vision with refraction was observed after the three-month follow-up.

## Discussion

LMBBS is a rare genetic disorder with significant clinical heterogeneity, and this case report presents a unique manifestation in a 10-year-old girl. The patient displayed a combination of features, including atypical retinitis punctata albescens and NAFLD, expanding the spectrum of clinical presentations associated with LMBBS. The characteristic ocular findings in LMBBS typically involve retinitis pigmentosa. However, the present case introduced an atypical presentation termed "retinitis punctata albescens," characterized by bilateral retinal involvement with a pale disc and arterial attenuation. This adds to the growing literature emphasizing the varied ocular phenotypes within the LMBBS spectrum [8].

The rarity of this specific retinal manifestation underscores the complexity of genetic interactions and molecular pathways involved in LMBBS. The genetic basis of LMBBS involves mutations in multiple genes, including those encoding for ciliary proteins, highlighting the importance of primary cilia in the development and function of various organs, including the retina [11]. Beyond ocular manifestations, LMBBS typically affects multiple organ systems. In this case, the patient exhibited central obesity, dysmorphic facial features, and polydactyly, aligning with the classical clinical features associated with LMBBS. Including NAFLD in this presentation adds to the understanding of the systemic involvement in LMBBS [12].

The dyslipidemia observed in our patient, with decreased HDL and LDL levels, is consistent with previous reports in LMBBS cases [4]. The association of NAFLD with LMBBS, however, is less commonly documented. This highlights the need for comprehensive monitoring and management of metabolic aspects in individuals with LMBBS, considering the potential long-term implications on overall health [13]. Consanguineous marriages have been associated with an increased risk of autosomal recessive disorders, including LMBBS. Genetic counseling played a crucial role in educating the parents about potential complications and guiding them through the diagnostic process [14]. The recommendation for genetic analysis aimed at the early detection of anomalies and enabling appropriate interventions aligns with existing literature emphasizing the importance of early diagnosis and intervention in improving outcomes for individuals with LMBBS [6]. The management approach in this case involved a multidisciplinary strategy, including corrective glasses, dietary modifications, vitamin supplementation, and exercise recommendations. Unfortunately, the lack of improvement in vision during the follow-up period highlights the challenges in managing the progressive nature of the ocular manifestations in LMBBS.

## Conclusions

This case report highlights the diverse clinical features of LMBBS in a 10-year-old girl, showcasing a rare combination of atypical retinitis punctata albescens, polydactyly, central obesity, and NAFLD. Despite comprehensive management efforts, the patient's visual impairment did not improve, underscoring the challenging and progressive nature of LMBBS, particularly its ocular manifestations. Genetic counseling played a pivotal role, emphasizing the importance of early genetic analysis in consanguineous marriages for anomaly detection and informed family planning decisions. This case contributes to our understanding of LMBBS and underscores the need for ongoing research and multidisciplinary care to address its complexities.
